# Acute necrotic disorder of the small intestine post‐coronavirus disease‐2019 vaccination

**DOI:** 10.1002/deo2.137

**Published:** 2022-06-05

**Authors:** Tomoyuki Nishimura, Seiji Onogawa, Takuya Yamamoto, Yasuhiro Okuda, Morito Ikeda, Nozomu Matsumoto, Keisuke Kurihara, Akinori Shimizu, Shosuke Kitamura, Yoshio Katamura, Naomichi Hirano, Shingo Itamoto, Masahiro Nakahara, Shuji Yonehara, Fumio Shimamoto, Keiji Hanada

**Affiliations:** ^1^ Department of Gastroenterology Onomichi General Hospital Hiroshima Japan; ^2^ Department of Surgery Onomichi General Hospital Hiroshima Japan; ^3^ Department of Pathology Onomichi General Hospital Hiroshima Japan; ^4^ Department of Nursing Faculty of Health Sciences Hiroshima Cosmopolitan University Hiroshima Japan

**Keywords:** COVID‐19, necrosis, side effects, small intestine, vaccines

## Abstract

The Pfizer‐BioNTech coronavirus disease 2019 (COVID‐19) vaccine is extensively used worldwide, and its safety has been proven. Herein, we report a case of an acute necrotic disorder in the small intestine post‐COVID‐19 vaccination. The patient developed severe abdominal pain the day after the first vaccination. Contrast‐enhanced computed tomography showed extensive ileum wall thickening and ascites. Colonoscopy revealed a ring‐shaped ulcer and stricture in the terminal ileum. Ileocecal resection was performed, and the patient did not have further episodes of a necrotic disorder in the small intestine. Although it is unknown if this event is associated with vaccination, and this occurrence also does not outweigh the efficacy and safety of the Pfizer‐BioNTech COVID‐19 vaccine, gastroenterologists need to be aware of this rare case, given its noteworthy timing.

## INTRODUCTION

The coronavirus disease‐2019 (COVID‐19) emerged in China in 2019 and rapidly spread worldwide. It was caused by the severe acute respiratory syndrome coronavirus 2 and resulted in multiple severe cases and deaths.^1^ On December 11, 2020, the US Food and Drug Administration granted emergency approval to Pfizer‐BioNTech's COVID‐19 vaccine, which was extensively administered worldwide. Pfizer's COVID‐19 vaccine uses a pioneering messenger RNA (mRNA) technology that has never been used previously in vaccine production, and several concerns arose regarding its side effects. However, the previously reported side effects rarely required hospitalization.^2^ In this report, we discuss a case of an acute necrotic disorder of the small intestine that required surgical resection in a patient after he received the first dose of the Pfizer‐BioNTech COVID‐19 vaccine.

## CASE REPORT

A 72‐year‐old male visited the emergency department complaining of acute‐onset abdominal pain. He had received his first dose of the Pfizer‐BioNTech BNT16B2b2 mRNA vaccine one day before visiting the hospital. He had a history of cerebral infarction and immunoglobulin G (IgG) 4‐related kidney disease. His current medications were clopidogrel (75 mg) and prednisolone (5 mg). He was conscious and afebrile. Physical examination revealed abdominal tenderness without rebound tenderness or guarding. He had decreased bowel sounds and normal vital signs. Laboratory testing revealed a slightly elevated white blood cell count of 14,400/μl (reference range, 3300–8600/μl). Other markers, including D‐dimer, were normal. He was admitted to the hospital for suspected enteritis.

**FIGURE 1:**
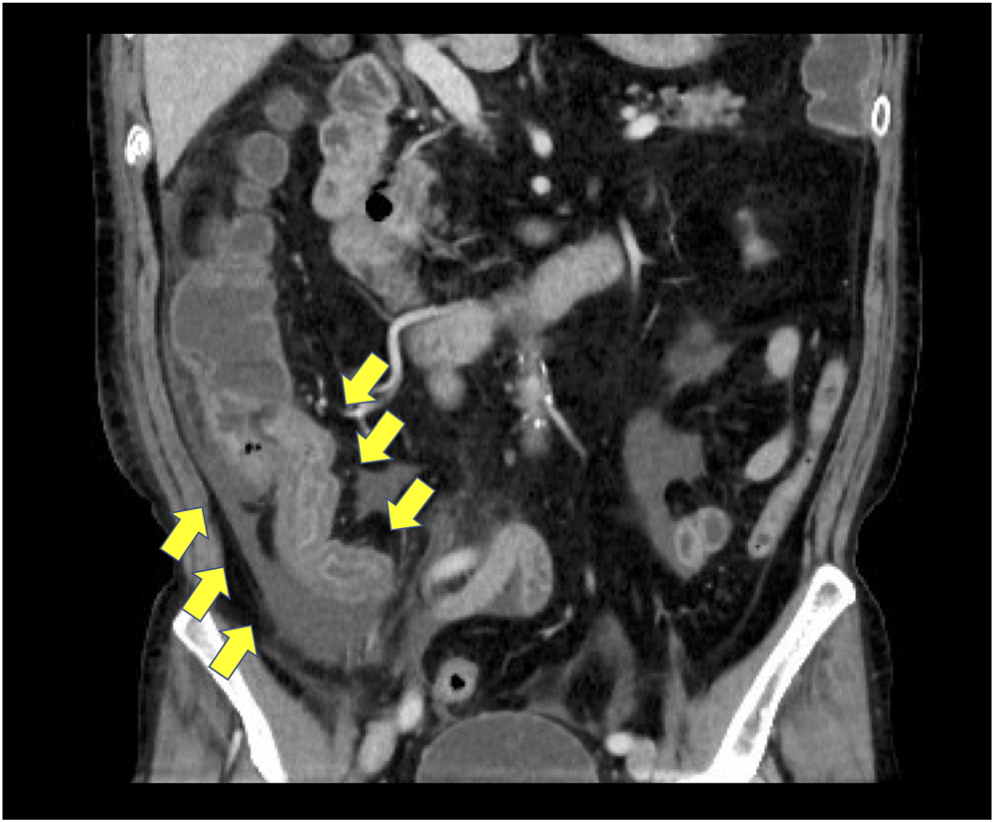
Contrast‐enhanced computed tomography showed that the terminal ileum wall was thickened, and ascites was observed around it. However, the contrast effect was relatively well preserved

**FIGURE 2:**
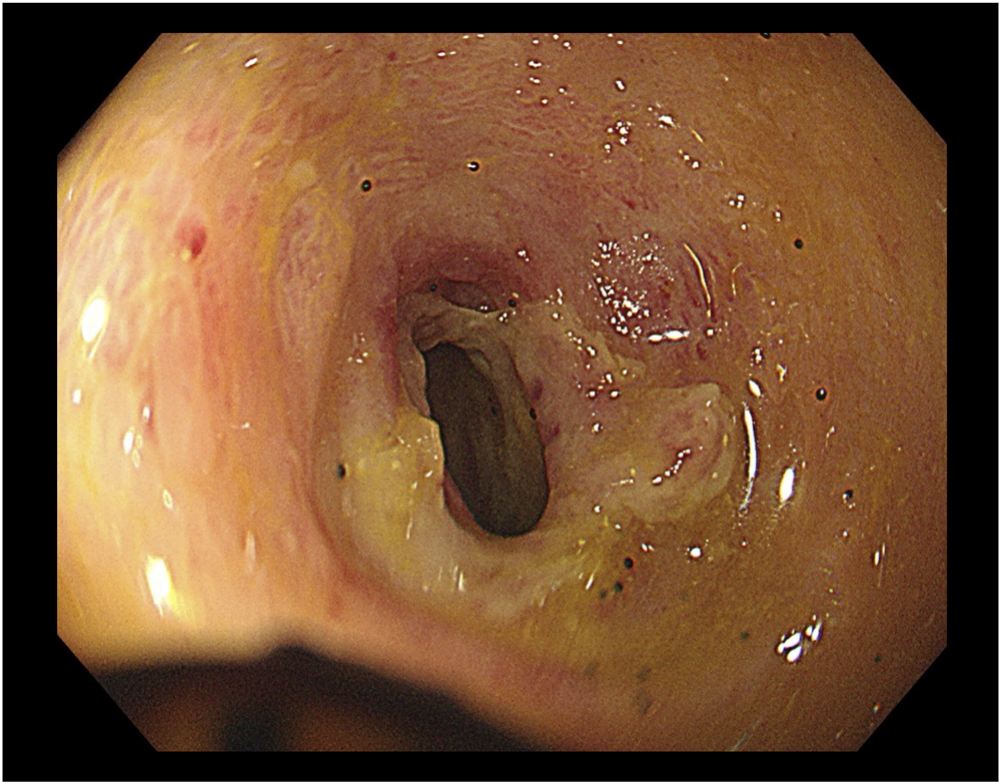
Colonoscopy revealed a ring‐shaped ulcer and stricture in the terminal ileum. The colonoscope did not pass into the small intestine on the oral side of the ulcer

**FIGURE 3:**
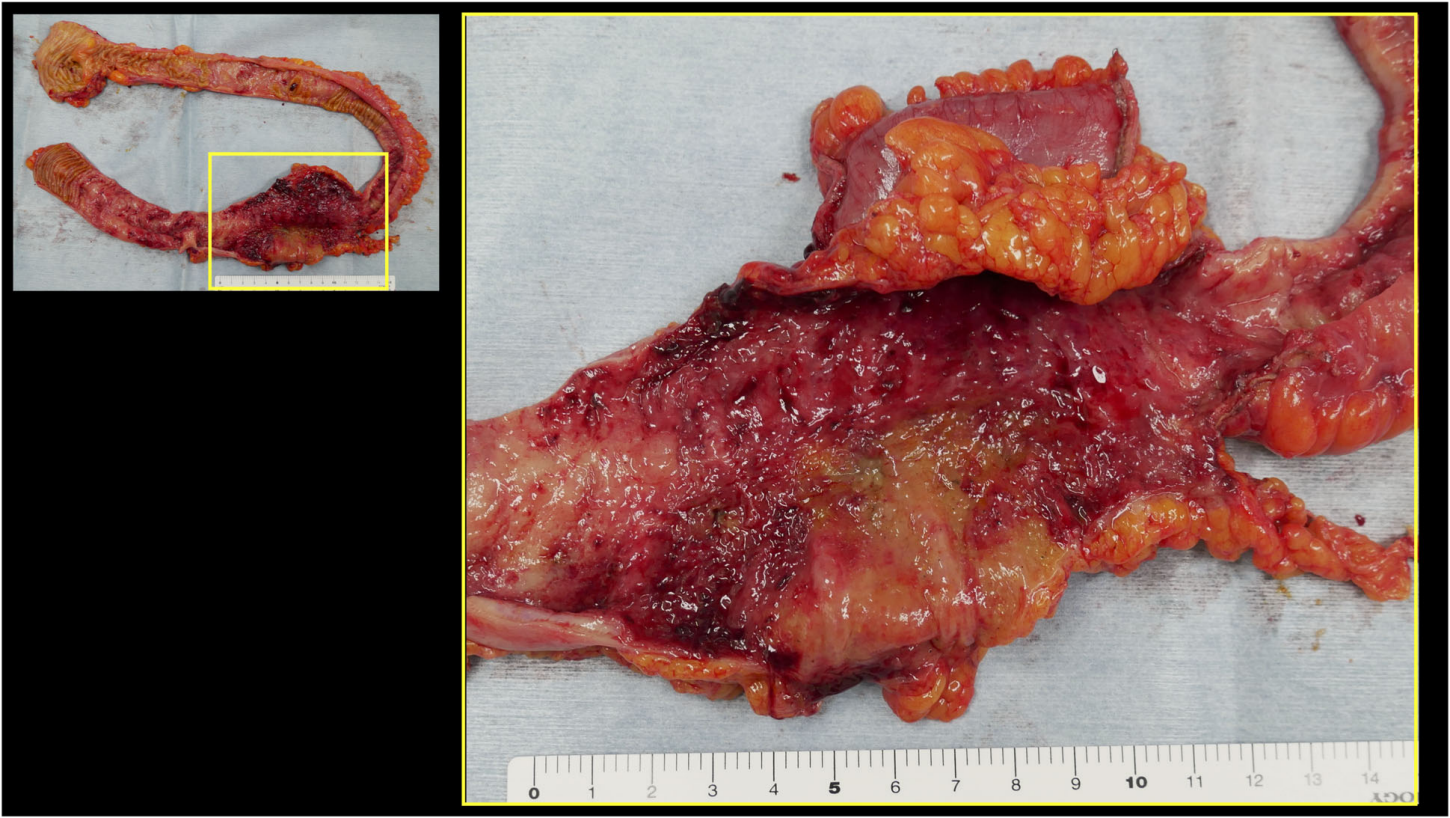
Ulceration and necrosis were observed in the ileocecal region

**FIGURE 4:**
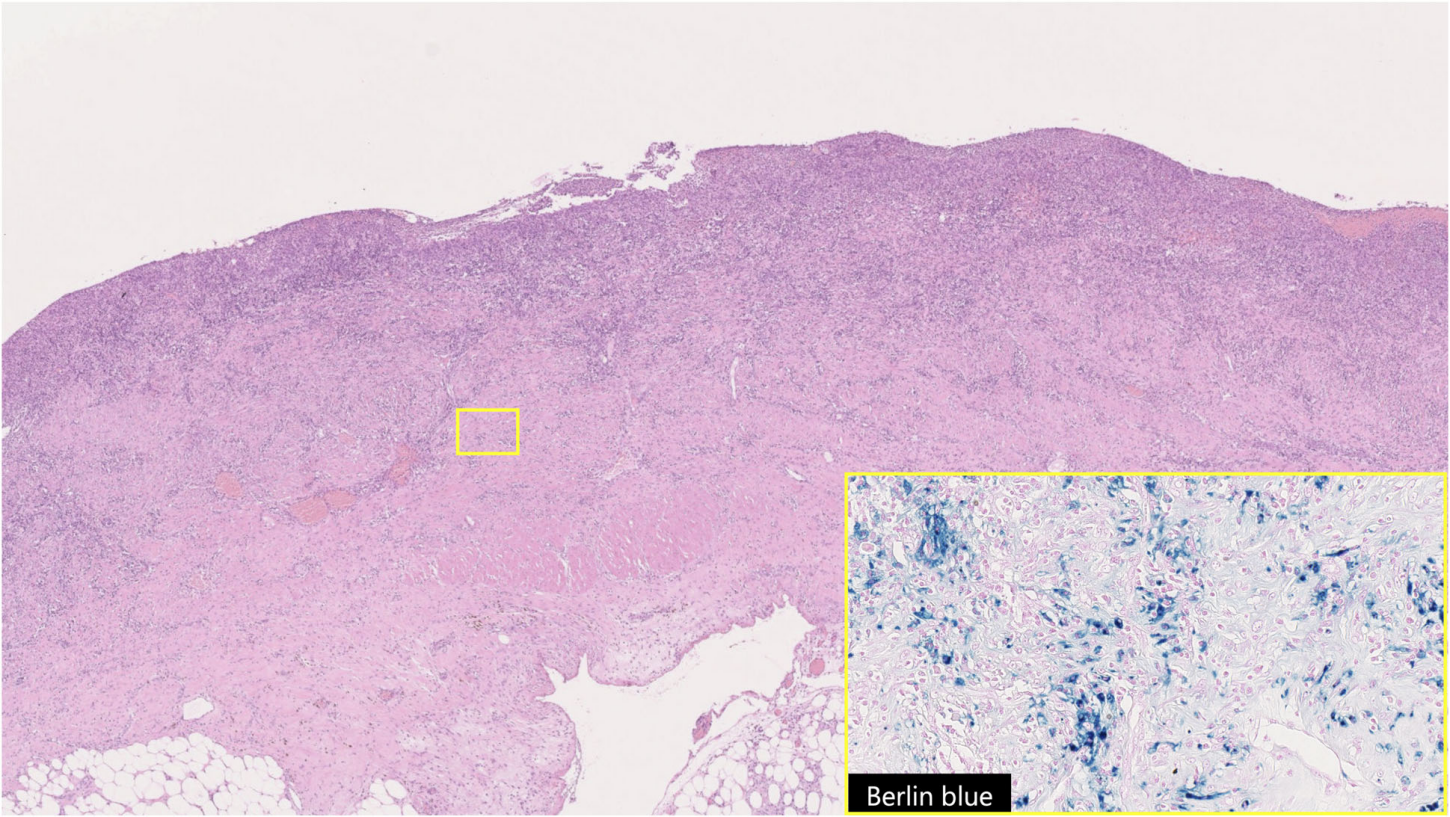
The laminar structure of the intestinal tract was replaced almost entirely by inflammatory granulation tissue. Berlin blue staining showed hemosiderin‐phagocytosed macrophages

One day after his admission, the abdominal pain worsened. His white blood cell count was elevated to 17,100/μl, C‐reactive protein level was 13.70 mg/dl (reference value, < 0.14 mg/dl), and D‐dimer level was 4.6 μg/ml (reference range, 0–1 μg/ml). Contrast‐enhanced computed tomography showed extensive ileum wall thickening and ascites; there were no abnormal findings in the vessels around the terminal ileum (Figure 1). These findings were likely manifestations of enteritis exacerbation. Thus, the patient was instructed to fast and was initially treated with broad‐spectrum intravenous antibiotics. However, as the symptoms failed to improve, IgG4‐related enteritis was suspected. Prednisolone (40 mg) was administered on day 4 of hospitalization, but it was ineffective. The abdominal pain became more severe, and he developed diarrhea and fever. Surgical resection of the bowel was considered; however, the patient refused it. Blood and stool cultures showed unremarkable results. Colonoscopy performed on day 32 revealed a ring‐shaped ulcer and stricture in the terminal ileum with normal findings in the large intestine (Figure 2). A colonoscopy performed one year ago showed normal findings in the terminal ileum. Small intestinal biopsy results excluded intestinal tuberculosis or cytomegalovirus enteritis, and the IgG4 infiltration was mild. A barium contrast X‐ray showed 60‐cm stenosis at the end of the ileum, which was dilated starting from the oral side. The patient was diagnosed with small intestinal obstruction due to necrotic enteritis, and ileocecal resection was performed on day 41. The mucosa of the excised ileum was thinned and partially necrotic (Figure 3). The laminar structure of the intestinal tract was replaced almost entirely by inflammatory granulation tissue. Berlin blue staining showed hemosiderin‐phagocytosed macrophages, indicating necrosis with ischemic changes.

(Figure 4). The findings were consistent with acute necrotic small bowel inflammation and few immunoglobulin G4‐positive cells were noted. Postoperatively, his fever and abdominal pain improved, and he was discharged on a postoperative day 14 without major complications. No new events have occurred since discharge.

## DISCUSSION

Among patients with COVID‐19, both arterial and venous thrombosis are reportedly noted in those with a strong thrombotic tendency. Specifically, cerebral infarction, myocardial infarction, and limb artery thrombosis have been reported as arterial thrombosis events, while deep vein thrombosis and pulmonary thromboembolism have been reported as venous thrombosis events.^3^ Additionally, prophylactic anticoagulant therapy has not been associated with a reduction in thrombosis incidence.^4^ In this case, severe necrotic small bowel inflammation occurred despite the use of anticoagulant medication.

Three vaccines have been approved in Japan: two mRNA vaccines, developed by Pfizer/BioNTech and Moderna, and the virus vector vaccine, developed by AstraZeneca. These vaccines do not contain a live virus. Although genetic information is inoculated into the body, it does not alter human genetic information. In addition, the mRNA is broken down within a few days after vaccination, and the spike protein produced disappears within two weeks after vaccination. Based on the mechanism of the mRNA vaccine, few adverse reactions are expected.

Mild, local, and systemic reactions have been reported in patients recently vaccinated against COVID‐19. According to Chapin‐Bardales et al., the most frequently reported local and systemic reactions after the first COVID‐19 vaccine dose were injection site pain (67.8%), fatigue (30.9%), headache (25.9%), and myalgia (19.4%). Reactogenicity was substantially greater after the second dose for both vaccines, particularly for systemic reactions, including fatigue (53.9%), headache (46.7%), myalgia (44.0%), chills (31.3%), fever (29.5%), and joint pain (25.6%).^5^ Abdominal pain was observed in 3.0% of patients after their first dose and in 6.1% after their second dose. However, severe thrombosis was not observed. In previous reports, most adverse events after COVID‐19 vaccination were mild or moderate.^6^ However, severe thrombosis with thrombocytopenia has also been identified as an adverse reaction. Since March 2021, several case series of unusual thrombotic events and thrombocytopenia have been reported in Germany, Norway, and the UK.^7^, ^8^, ^9^ However, there have been no reports of small intestinal disorders after COVID‐19 vaccination. On April 7, 2021, the European Medicines Agency concluded that the condition should be listed as an "infrequent adverse reaction, considered similar to heparin‐induced thrombocytopenia. Tarawneh et al. reported that a 22‐year‐old healthy man developed severe thrombocytopenia three days after receiving the Pfizer‐BioNTech BNT16B2b2 mRNA vaccine.^10^ In our case, thrombocytopenia did not occur. However, findings, such as an elevated D‐dimer level, suggested necrotic disorder. This was a rare case of severe necrotic disorder that developed immediately after vaccination. However, it is unknown if this event is associated with vaccination. There is a possibility of an accidental complication. This occurrence also does not outweigh the efficacy and safety of the Pfizer‐BioNTech BNT16B2b2 mRNA vaccine. We believe that reporting this case is important to create awareness among clinicians to watch for and report similar cases for the accumulation of data.

## CONFLICT OF INTEREST

The authors declare that they have no conflict of interest.

## FUNDING INFORMATION

None.

## ETHICS STATEMENT

Not applicable.
